# Harnessing Mediterranean Halophytes as Sustainable Bio-Factories: Tackling Microbial Challenges Across the Agrifood Chain

**DOI:** 10.3390/molecules31213953

**Published:** 2026-11-02

**Authors:** Panayiota Piri, Atalanti Christou, Georgia D. Ioannou, Vlasios Goulas, George Botsaris

**Affiliations:** Department of Agricultural Sciences, Biotechnology and Food Science, Cyprus University of Technology, Lemesos 3603, Cyprus; ps.piri@edu.cut.ac.cy (P.P.); atalanti.christou@cut.ac.cy (A.C.); georgia.ioannou@cut.ac.cy (G.D.I.)

**Keywords:** halophytes, Mediterranean basin, antimicrobial, antifungal, phytochemicals, agrifood production systems

## Abstract

The Mediterranean basin is a global biodiversity hotspot characterized by a high frequency of halophytic flora, which have evolved specialized metabolic adaptations to thrive under hypersaline, drought, and high-UV conditions. These physiological mechanisms facilitate the biosynthesis of diverse secondary metabolites, which have garnered significant attention for their antimicrobial and antifungal properties. This review critically evaluates the antimicrobial potential of nine prominent Mediterranean halophytes, namely *Cakile maritima*, *Crithmum maritimum*, *Eryngium maritimum*, *Juncus acutus*, *Juncus maritimus*, *Limbarda crithmoides*, *Otanthus maritimus*, *Pancratium maritimum*, and *Salicornia europaea*, synthesizing data on their phytochemical profiles and efficacy against foodborne, clinical, and agricultural pathogens. The findings indicate that several extracts and essential oils exhibit potent inhibitory effects, with minimum inhibitory concentration (MIC) values often comparable to or even lower than those of established antibiotics, notably against *Staphylococcus* spp., *Salmonella* spp., and various *Candida* species. These halophyte extracts are also used successfully to control microbial contamination in the production of plant- and animal-based foods. Despite the significant potential for agricultural and food sectors, full commercialization requires addressing challenges related to field-level efficacy, formulation stability, and comprehensive toxicological safety. Overall, this study underlines the role of Mediterranean halophytes as resilient bio-factories capable of providing sustainable, natural solutions to pressing antimicrobial resistance and food security challenges.

## 1. Introduction

Halophytes constitute a specialized group of salt-tolerant flora, representing approximately 1% of global terrestrial plant diversity across roughly 550 genera and 120 families [1]. Formally defined by their capacity to complete their entire ontogenetic life cycle in substrates exceeding 200 mM NaCl, these plants thrive within hostile coastal zones, mangroves, and salt marshes where they frequently tolerate hypersaline conditions, high ultraviolet radiation, and drought stress [2]. In addition to mitigating osmotic and ionic stress, exposure to specific salinity thresholds promotes halophytic biometric development by increasing leaf surface area and boosting photosynthetic capacity [3]. This physiological resilience is governed by a synergistic combination of cellular and anatomical adaptations. Principal among these mechanisms are the active transport and vacuolar storage of toxic Na^+^ and Cl^−^ ions, the accumulation of organic solutes in the cytoplasm to maintain cell turgor, and the active excretion of excess salts through highly selective salt glands in the epidermis [4]. Consequently, their unique metabolic responsiveness has positioned halophytes as a valuable reservoir of novel bioactive secondary metabolites with significant potential for food, cosmetic and pharmaceutical applications [5,6].

As a globally recognized biodiversity hotspot, the Mediterranean basin harbors between 15,000 and 25,000 plant species, approximately 60% of which are endemic, a profound floristic diversity driven by complex geomorphology and varied microclimatic conditions [7]. A significant component of this native flora comprises halophytes, which are uniquely characterized by the accumulation of bioactive secondary metabolites and essential nutrients. In recent decades, environmental pressures have elevated the ecological relevance of these species. The region is highly sensitive to anthropogenic climate change, which has exacerbated seawater intrusion into coastal aquifers, increased soil salinization, and constrained freshwater availability for irrigation. Consequently, the Mediterranean basin now ranks among the most salinity-affected regions globally [7,8].

Halophytes are a rich source of bioactive compounds, exerting a wide spectrum of pharmacological effects on human health, with their potent antimicrobial activity garnering particular attention. The antimicrobial efficacy of halophyte-derived extracts holds profound implications for food preservation, where microbial spoilage remains an unresolved socioeconomic challenge despite the implementation of contemporary technological interventions [9]. In addition, the global proliferation of multidrug-resistant pathogens underscores an urgent demand for novel therapeutic scaffolds, justifying the exploration of halophytic flora for the development of innovative antibacterial and antimycotic agents [10].

**Figure 1:**
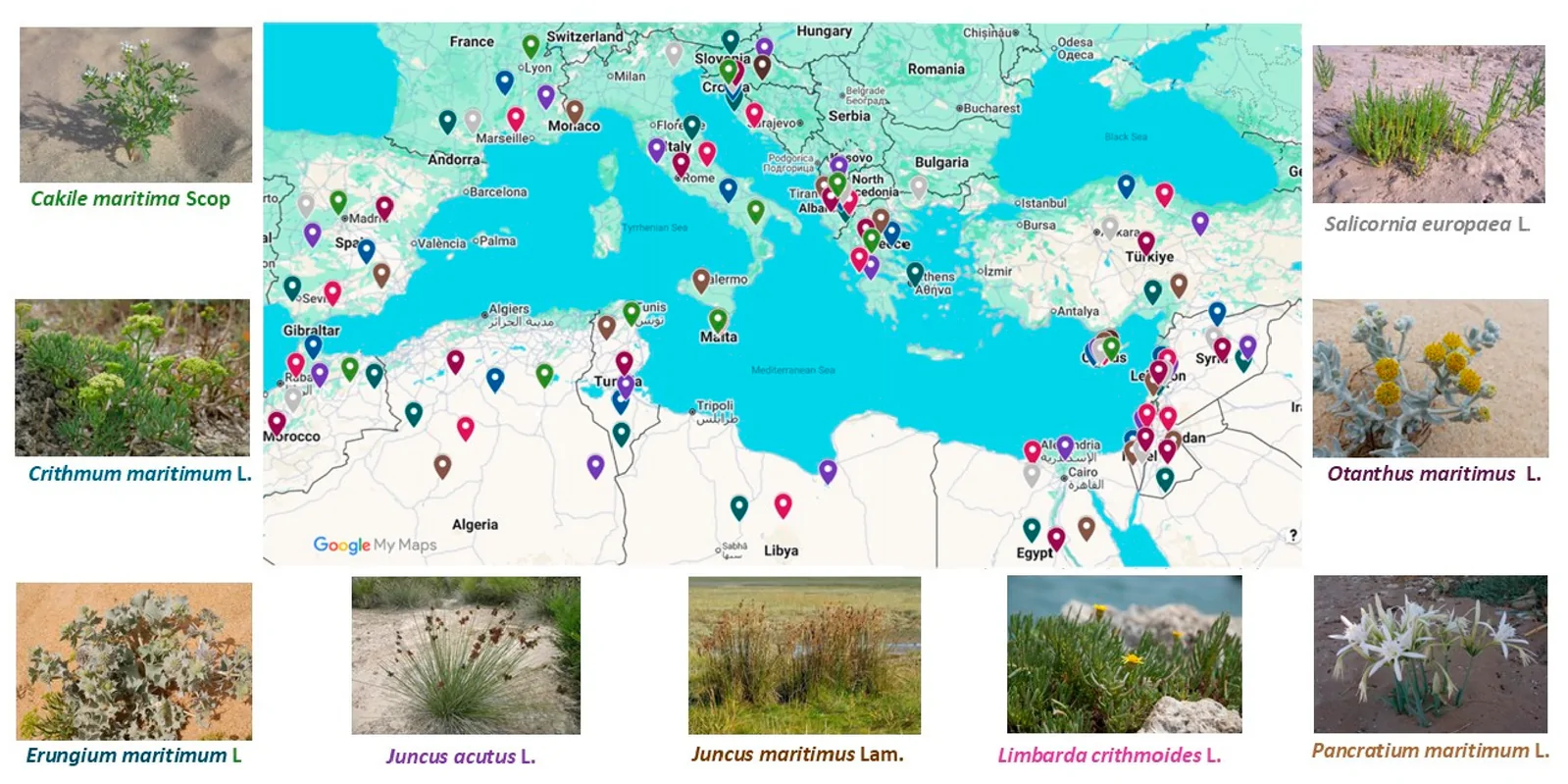
Geographic distribution map of studied Mediterranean halophytic plant species.

To provide a comprehensive assessment, this review focuses on nine selected Mediterranean halophytic species belonging to six different plant families (Figure 1). Specifically, *Salicornia europaea* L., a member of the most dominant halophytic group (Amaranthaceae) in the Mediterranean, is extensively documented for its exceptional salinity tolerance, nutritional value, and pharmacological potential [11]. *Crithmum maritimum* L. and *Eryngium maritimum* L. are prominent halophytic coastal perennials native to Mediterranean maritime habitats of the Apiaceae family, widely recognized for their ecological resilience, rich phytochemical profiles, and traditional medicinal applications [12,13]. In addition, *Juncus acutus* L. and *Juncus maritimus* Lam. accumulate diverse bioactive phenanthrenes and polyphenols with pronounced antibacterial and antifungal capacity [14]. Members of the Asteraceae family, *Limbarda crithmoides* L. and *Otanthus maritimus* L., are characterized by essential oils, sesquiterpenes, and polyphenols with substantial biological activity [15,16,17,18]. Finally, *Cakile maritima* Scop. (Brassicaceae) and *Pancratium maritimum* L. (Amaryllidaceae) synthesize specialized metabolites, most notably glucosinolates and Amaryllidaceae alkaloids with remarkable inhibitory effects on food and plant pathogens [5,19]. In general, these halophytes act as “green” bio-factories accumulating different classes of bioactive phytochemicals, which exhibit robust inhibitory effects against clinical, foodborne, and agricultural pathogens. Consequently, this review aims to critically evaluate the antimicrobial and antifungal potential of selected Mediterranean halophytes, synthesizing accumulated evidence regarding their biological activity. To elucidate the mechanisms underlying the inhibitory effects of halophytic extracts and essential oils, their bioactive phytochemical profiles are systematically documented. Furthermore, this review documents the practical applications of halophyte-derived antimicrobials within the food production chain.

## 2. Bioactive Composition of Halophytes

**Figure 2:**
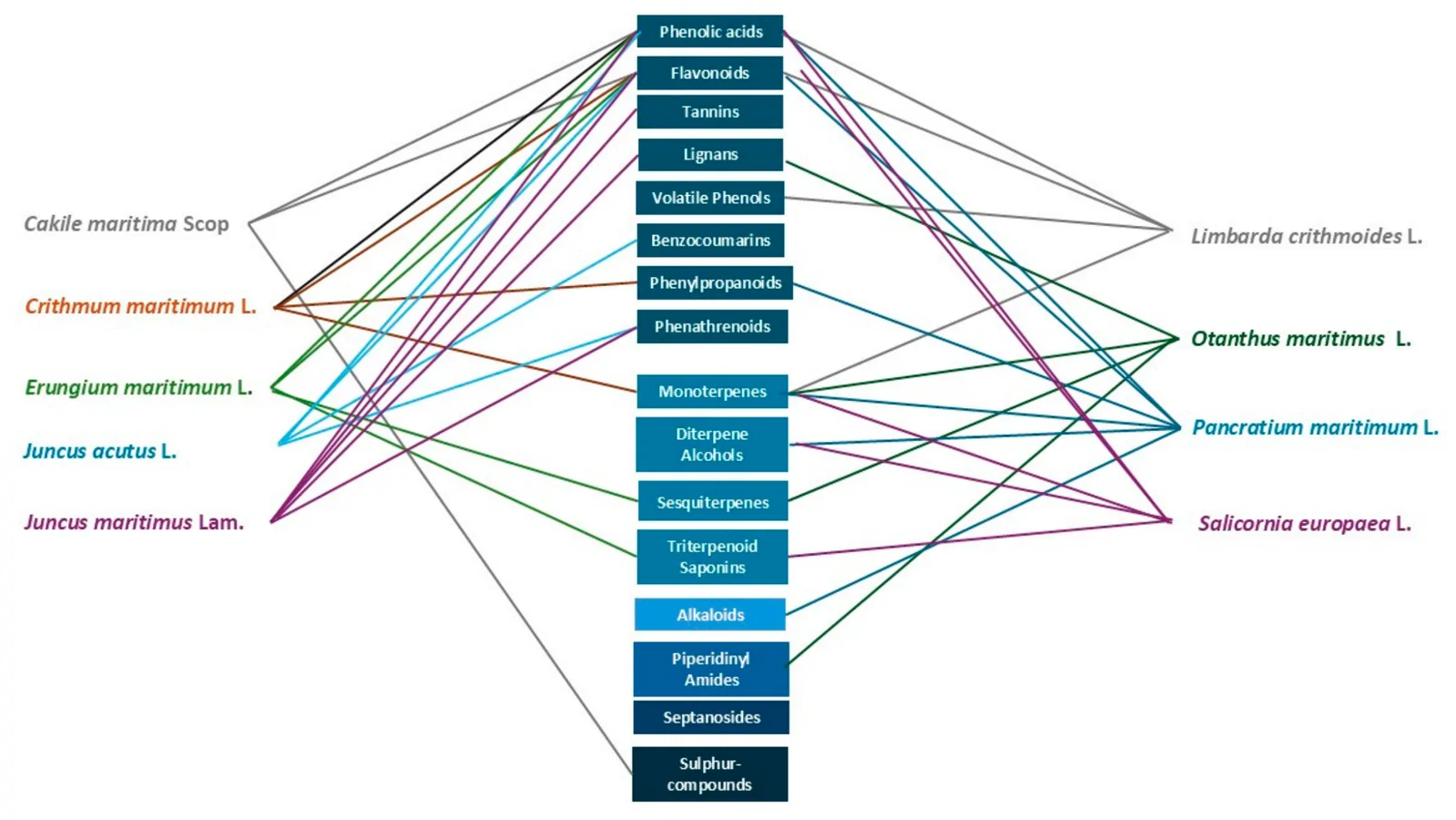
Phytochemical profiles of the nine selected halophytes, displaying the distribution of secondary metabolite classes across each species.

Figure 2 illustrates the distribution of major phytochemical classes across a selection of coastal halophytes, highlighting both shared and species-specific metabolic profiles. Phenolic compounds, including phenolic acids, flavonoids, tannins, lignans, and phenylpropanoids, are broadly represented across most taxa, indicating their fundamental role in stress adaptation in saline environments. Terpenoids, such as monoterpenes, sesquiterpenes, diterpene alcohols, and triterpenoid saponins, exhibit a more heterogeneous group, with species like *Otanthus maritimus* and *Pancratium maritimum* showing particularly diverse terpenoid structures. Nitrogen- and sulfur-containing metabolites, including alkaloids, piperidinyl amides, and sulfur compounds, appear more restricted and species-dependent, reflecting specialized biosynthetic capabilities.

*Cakile maritima* Scop. (Brassicaceae; Sea rocket)

The phenolic profile of *C. maritima* is characterized by its diversity and organ-specific distribution, which is largely influenced by environmental adaptations, such as salt stress that triggers the phenylpropanoid biosynthetic pathway. Leaves exhibit the highest concentration of phenolics within the plant, characterized by a substantial abundance of benzoic and cinnamic acids, as well as specialized phenolic choline esters and flavonol glucosides. Conversely, seeds and flowers exhibit differing accumulation patterns. Flowers demonstrate the lowest overall phenolic concentration, while seeds possess a moderate yield but they are exceptionally enriched in phenolic acids, most notably chlorogenic acid, ellagic acid, and caffeic acid [5,20]. Furthermore, *C. maritima* contains a wealth of volatile sulfur-containing compounds, including ten isothiocyanate derivatives that are characteristic of the Brassicaceae family [21].

*Crithmum maritimum* L. (Apiaceae; Sea fennel)

*C. maritimum* L. is characterized by a complex biochemical profile rich in hydrophilic and lipophilic bioactive constituents, including phenolic acids, flavonoids, carotenoids, vitamins, and volatile essential oils [13]. Leaves, stems, and flowers serve as a potent source of phenolic compounds, depending on the plant part and the extraction methodology employed. Chlorogenic acid is the predominant phenolic constituent, accounting for 30–50% of the total fraction. Other major polyphenols include caffeic acid, rutin, and quercetin, alongside trace concentrations of ferulic, gallic, and sinapic acids [22,23].

The essential oil fraction of *C. maritimum* is primarily composed of monoterpenes and phenylpropanoids, which dictate their antimicrobial properties. The volatile profile exhibits substantial qualitative and quantitative diversity driven by geographic origin, harvest season, and plant organ. Environmental and geographical factors drive distinct regional chemotypes. In Croatian, French, and Turkish populations, a sabinene/limonene-rich chemotype dominates, featuring 42–55% sabinene and 36–58% limonene. A dillapiole-rich chemotype is found in France (up to 75%), Bulgaria, and Greece (34–48%). Lastly, a γ-terpinene/thymyl methyl ether-rich chemotype characterizes Italian and Cypriot profiles. Minor volatiles across these regions include β-pinene, p-cymene, myrcene, carvone, terpinen-4-ol, and α-pinene [24,25,26,27].

*Erungium maritimum* L. (Apiaceae; Sea holly)

The phytochemical profile of *E. maritimum* is characterized by a diverse array of bioactive secondary metabolites, with hydroxycinnamic acid derivatives emerging as the major class. Among these, rosmarinic acid is found in the highest concentration, alongside other remarkable constituents such as neochlorogenic acid, chlorogenic acid, and feruloyl derivatives [28,29]. The existing literature confirms several flavonoids in this species, mostly consisting of glycosylated derivatives of kaempferol, quercetin, luteolin, and isorhamnetin [28,29]. Furthermore, it is recognized as a unique source of triterpene saponins; isolated saponins typically feature hydroxylated oleanane-type aglycones, with A1-barrigenol, R1-barrigenol, and barringtogenol C representing the primary structures [30]. Finally, the essential oil composition of this halophyte is marked by a high abundance of oxygenated sesquiterpenes, which account for approximately 55% of the total volatile metabolites. While quantitative variations in essential oil profiles are influenced by the plant’s geographical origin and the specific tissues analyzed, spathulenol, germacrene D, and amorpha-4,9-dien-2-ol consistently remain the major constituents [12,31].

*Juncus acutus* L. (Juncaceae; Sharp rush)

*J. acutus* L. is recognized as a significant source of nitrogen-free alkylated phenanthrenoids, a chemical class characteristic of this genus. These compounds exist as both monomeric and dimeric forms and exhibit diverse substitution patterns, including hydroxy, methoxy, alkyl, formyl, and carboxyl groups. To date, over 30 different phenanthrenoid structures have been identified within *J. acutus* [32]. Furthermore, investigations of the species’ rhizomes have led to the isolation of novel dimeric phenanthrenoids, as well as seven benzocoumarins, which are likely products of the oxidative transformation of existing phenanthrenes [33,34]. In addition to these constituents, *J. acutus* possesses a broad spectrum of phenolic compounds, including various phenolic acids, flavonoids, and [35,36].

*Juncus maritimus* Lam. (Juncaceae; Sea rush)

Similar to *J. acutus*, *J. maritimus* represents a promising source of phenanthrenes, characterized by a high degree of structural diversity [14]. Among these, juncunol, effusol, and juncusol have been identified as the most abundant 9,10-dihydrophenanthrenes in its fruits [37]. Furthermore, Kúsz et al. (2021) isolated and characterized four novel phenanthrene structures from the aerial parts of this halophyte collected in Croatia [38].

Regarding its phenolic composition, polar extracts of *J. maritimus* comprise phenolic acids, flavonoids, and tannins, with phenolic acids serving as the predominant class in both stems and fruits [37,39]. The halophyte also contains a significant amount of lignans; specifically, the monolignols coniferyl alcohol, sinapyl alcohol, and guaiacylglycerol, alongside ferulic acid, are linked to form cross-coupled dimeric products. Finally, dehydroabietic acid, an abietic-type diterpene, has been reported in minor quantities within the stems [37].

*Limbarda crithmoides* L. (Asteraceae; Golden samphire)

Phytochemical analyses of its aerial parts have identified a rich profile of phenolic acids and flavonoids, most notably chlorogenic, syringic, ferulic, and gallic acids, as well as rutin and various quercetin derivatives. Notably, Brzovic et al. (2025) identified 12 phenolic acids and six flavonoids within the plant’s hydroalcoholic extract [15]. The phenolic fraction is primarily composed of caffeoylquinic acid derivatives; specifically, chlorogenic acid (3-caffeoylquinic acid) and 1,5-di-caffeoylquinic acid have been reported as the most prominent constituents in Croatian and Tunisian populations, respectively [15,40]. Furthermore, analysis of the lipophilic extract has confirmed the presence of two thymol derivatives, representing a class of volatile phenols [41].

The essential oil of *L. crithmoides* is characterized by a terpene-rich profile, with 40 compounds accounting for 90% of its composition, primarily monoterpene hydrocarbons (45%) and oxygenated monoterpenes (40%), the latter dominated by thymol [16,42]. Comparative studies reveal that while monoterpene hydrocarbons consistently predominate across various Mediterranean populations, the overall profile is strongly influenced by geographical origin. Notably, β-phellandrene, limonene, and β-myrcene have been identified exclusively in specific Mediterranean subpopulations [42,43].

*Otanthus maritimus* L. (Asteraceae; Cotton weed)

The antimicrobial properties of *O. maritimus* L. have been definitively associated with its essential oil composition. Although the total phenolic content of specimens collected along the Iberian Peninsula has been reported to reach 5.54 mg g^−1^ fresh weight, the phenolic profile remains unexplored [44]. While the essential oil composition of O. *maritimus* exhibits significant variability across different geographic regions and harvest periods, the existing literature consistently identifies a terpene-rich profile predominantly composed of oxygenated mono- and sesquiterpenes. Comparative studies highlight this chemical diversity, revealing that populations from Corsica and Sicily are dominated by yomogi alcohol, artemisia alcohol, artemisyl acetate, and camphor [17,45]. Similarly, material collected in Greece is characterized by santolina triene, camphor, and artemisyl acetate [46]. Research on Algerian populations also revealed a distinct chemotype enriched in α-isocomene, β-isocomene, and α-epi-bisabolol [47]. Consequently, *O. maritimus* does not possess a uniform essential oil profile; rather, its chemical composition is highly contingent upon geographical and temporal factors, a critical consideration for any systematic review of its phytochemical profile. Furthermore, a series of piperidinyl amides of unknown antimicrobial efficacy was identidied, alongside the bioactive lignans sesamin and pinoresinol, both of which demonstrate potent antibacterial activity [18,48,49].

*Pancratium maritimum* L. (Amaryllidaceae; Sea daffodil)

Several alkaloids have been isolated from *P. maritimum* L., representing all established skeletal types of Amaryllidaceae alkaloids, including the lycorine, homolycorine, galanthamine, haemanthamine, crinine, tazettine, phenanthridone, phenanthridine, montanine, and norbelladine classes [50]. Furthermore, it exhibits a broad diversity of phenolic compounds, specifically hydroxycinnamic acids and flavonoids. Hydroxycinnamic acids occur in both free forms and conjugates with sugars or amides. Among the flavonoids, glycosylated flavonols, primarily derivatives of quercetin, kaempferol, and isorhamnetin, are the most abundant; flavonol-C-glucosides and flavanones have also been detected [51]. Recently, the chemical composition of the essential oil from its aerial parts was characterized for the first time. The oil comprises thirteen groups of volatile compounds, with alcohols constituting nearly 40% of the total composition. Notably, the essential oil contains phenylpropanoids, monoterpenes, diterpene alcohols, and sesquiterpenes, all of which are recognized for their antimicrobial properties [52].

*Salicornia europaea* L. (Amaranthaceae; Marsh samphire)

*S. europaea* is a halophytic species recognized for its extensive array of bioactive secondary metabolites, which exhibit significant qualitative and quantitative diversity. The plant’s phytochemical profile is mostly defined by oleanane-type triterpenoid saponins, a prominent class represented by over twenty identified structures. Concurrently, caffeoylquinic acid derivatives constitute an essential group; recent investigations over the past two decades have elucidated various novel structures within this class, characterized by the presence of one or two caffeoyl moieties alongside various methyl and hydroxyl substitutions. Beyond these constituents, *S. europaea* contains a diversity of flavonoids, lignans, and chromones, which have been frequently correlated with diverse therapeutic activities and health-promoting properties [53,54]. The volatile fraction of this species remains largely uncharacterized, although research has identified the presence of six classes of organic compounds: namely terpenes, diterpenes, alkanes, alkenes, phenols, and fatty acids [55].

## 3. Antibacterial Activity of Halophytes

The antibacterial activity of selected halophytes from the Mediterranean region is summarized in Table 1. MIC values are presented to facilitate direct comparison of antimicrobial potency among the studied species. As shown in Table 1, polar and non-polar extracts, as well as essential oils, exhibit a wide range of inhibitory effects against both Gram-positive and Gram-negative bacteria. The antimicrobial efficacy is markedly influenced by several factors, including plant material, extraction method, extraction solvent, and the targeted bacterial strains. Consequently, MIC values for plant extracts and essential oils range from 1 μg mL^−1^ to 50 mg mL^−1^. The present comprehensive review provides a detailed description of halophytes with MIC values lower than 1000 μg mL^−1^ since higher MIC values indicate rather weak antimicrobial potency. A comprehensive analysis of the antimicrobial properties of individual halophyte species is provided in the following sections.

*Cakile maritima* Scop.

The antimicrobial activity of sea rocket remains relatively underexplored; however, early investigations by Meot-Duros (2008) demonstrated notable antibacterial effects of leaf-derived extracts, with reported MIC values at 1 μg mL^−1^ [56]. In that study, non-polar extract exhibited greater antimicrobial efficacy than polar ones. Specifically, non-polar extract inhibited nine microbial strains, whereas polar extracts were effective against only two. *Listeria monocytogenes* was resistant to all tested extracts, while *Pseudomonas aeruginosa* and *Pseudomonas fluorescens* showed the highest susceptibility. Notably, growth inhibition of *P. aeruginosa* was achieved with only 1 μg mL^−1^ of the polar fractions. For most tested microorganisms, MIC values were approximately 100 μg mL^−1^, highlighting the promising antibacterial potential of this species [59]. Further insights were provided by Fuochi et al. (2019), who evaluated the antimicrobial properties of ethanolic extracts obtained from different plant parts (leaves, flowers, seeds, and stems) of sea rocket [20]. Among these, only seed extracts exhibited significant antibacterial activity against a panel of clinically relevant strains, including *Staphylococcus aureus*, *P. aeruginosa* ATCC 27853, *Escherichia coli*, *Enterococcus faecalis*, *Acinetobacter baumannii*, and *Klebsiella pneumoniae*. The MIC values for *E. coli* and *P. aeruginosa* were 75 μg mL^−1^, whereas higher MIC values of 150 μg mL^−1^ were recorded for *S. aureus*, *E. faecalis*, *K. pneumoniae*, and *A. baumannii*. These findings indicate that antimicrobial efficacy in *C. maritima* is strongly dependent on plant part and extraction conditions, with seeds representing the most bioactive tissue. Regarding bioactive composition, ellagic acid and hydroxybenzoic acids, namely caffeic acid, chlorogenic acid and fertaric acid, are the major constituents of ethanolic extract of seeds [20].

*Crithmum maritimum* L.

Despite *C. maritimum* L. containing a complex matrix of polar and apolar phytoconstituents known for their potential nutritional and health-promoting benefits, research attempts have disproportionately favored its essential oils over extracts. While the literature frequently highlights the antimicrobial capacity of essential oils, many early studies relied on preliminary screening assays or utilized exceptionally high concentrations (1000–10,000 μg mL^−1^). Nevertheless, some evidence underscores a significant antimicrobial profile for sea fennel EOs; for instance, uniform sensitivity across various strains (*S. aureus, Listeria innocua, E. coli*) has been noted at a MIC of approximately 110 µg mL^−1^, with activity often attributed to major components such as thymol methyl ether, gamma-terpinene, and *p*-cymene [58]. Similar potent effects have been observed against *Campylobacter jejuni* at 300 μg mL^−1^ [60].

In contrast, findings regarding hydroethanolic extracts of sea fennel are inconsistent. Several studies report negligible inhibitory effects against a broad panel of Gram-negative and Gram-positive bacteria, with MIC values exceeding 2500 μg mL^−1^ [22,66]. Conversely, other research indicates that hydroethanolic extracts can significantly inhibit bacterial growth, including biofilm formation in *S. aureus*, in a dose-dependent manner, even at concentrations as low as 250 μg mL^−1^ [58]. Furthermore, investigations into non-polar extracts (e.g., petroleum ether and acetone) have demonstrated superior efficacy, particularly from flower and schizocarp tissues, yielding an MIC value of 75 μg mL^−1^ against *S. aureus*, while leaf extracts proved notably less effective [57].

*Eryngium maritimum* L.

The apolar extract of *E. maritimum* roots demonstrated notable antimicrobial activity against a range of bacterial species. Susceptibility varied by species, with MICs ranging from 78 μg mL^−1^ for *Stenotrophomonas maltophilia* to 1250 μg mL^−1^ for *Acinetobacter baumannii* and *Streptococcus dysgalactiae*. In contrast, the methanolic root extract showed no inhibitory activity against the tested human pathogens. Similarly, apolar organic extracts derived from aerial parts exhibited antimicrobial activity, albeit with higher MIC values (312–1250 μg mL^−1^) than those observed for the root extracts across a panel of Gram-positive and Gram-negative bacteria. On the contrary, the polar methanolic extract of the aerial parts displayed no antimicrobial efficacy at the tested concentrations. The antimicrobial activity of the apolar extracts has been largely attributed to their volatile constituents, primarily germacrene D and 8-hydroxy-cycloisolongifolene, followed by δ-guaiene, spathulenol, and palustrol [61]. Consistent with these findings, Meot-Duros et al. (2008) reported potent antimicrobial activity of apolar extracts against *B. aureus*, *S. aureus*, and *Salmonella* and *Pseudomonas* species, with MIC values ranging from 1 to 100 μg mL^−1^ [56]. Authors also observed that the methanolic extract of aerial parts exerted strong antimicrobial effects against *P. aeruginosa* and *P. fluorescens* at concentrations of 1 μg mL^−1^ [56]. Despite the remarkable efficacy of the apolar extracts, the essential oils derived from leaves and fruits exhibited high MIC values (>5000 μg mL^−1^), suggesting that their use as antimicrobial agents is not promising [67,68].

*Juncus acutus* L. and *Juncus maritimus* Lam.

The dichloromethane extracts of the whole plants of *J. acutus* and *J. maritimus* have been reported to exert significant antimicrobial activity against methicillin-resistant *S. aureus* (MRSA), with a MIC of 156 μg mL^−1^ and 78 μg mL^−1^, respectively. This capacity is mainly attributed to the presence of phenanthrenes, specifically juncusol and jinflexin B [62]. These findings align with earlier research on *Juncus* species, which indicated that apolar extracts derive the majority of their antibacterial efficacy from these specific compounds [64]. Furthermore, *J. maritimus* rhizome extracts have demonstrated strong-to-moderate antimicrobial activity against Gram-positive bacteria, such as *Streptococcus* and *Staphylococcus* species, though they remain inactive against a panel of six Gram-negative bacteria [63]. In contrast, ethanolic extracts from the roots and leaves, which are rich in hydroxycinnamic acids and flavonoid glycosides, exhibit only weak inhibitory effects against *S. aureus*, *P. aeruginosa*, and *Micrococcus luteus*, necessitating concentrations exceeding 2% *v/v* [36].

*Limbarda crithmoides* L.

The antimicrobial properties of *L. crithmoides* essential oils and extracts have been partially characterized. Evidence suggests that the essential oils possess high antimicrobial potency, with MIC values against *E. coli* and *S. aureus* reported below 6.25 μg mL^−1^, a threshold lower than that of the established antibiotic ciprofloxacin [64]. Recently, Jallali et al. (2026) also reported that the antimicrobial activity of *L. crithmoides* essential oils is highly variable, depending on the plant organ, geographical origin, and specific bacterial strain properties [16]. In this study, 30% of the samples demonstrated high-to-very-high activity, while 28% exhibited moderately high activity [16]. Notably, *Serratia marcescens* was the most sensitive strain, whereas *K. pneumoniae* showed the highest resistance. Furthermore, essential oils derived from leaves and flowers exhibited stronger antibacterial potential than those from roots and stems. Principal component analysis correlated this enhanced activity with the synergistic combination of mono- and sesquiterpenes [16]. Additionally, the antimicrobial activity of its extracts rich in phenolic compounds has been documented, with effects more pronounced against Gram-positive bacteria than Gram-negative ones. These extracts exhibited high-to-very-high antibacterial activity against *E. coli*, *Salmonella typhimurium*, *E. faecalis*, and *S. aureus*, with activity levels significantly influenced by the source plant organ. This strong efficacy has been attributed to the presence of 1,5-di-*O*-caffeoylquinic acid, a phenolic acid with well-established antimicrobial potency [40,69].

*Otanthus maritimus* L.

The antibacterial efficacy of essential oils derived from *O. maritimus* flowers has been evaluated against various Gram-positive and Gram-negative bacterial strains. The oils demonstrated remarkable inhibitory activity, with MICs ranging from 7.8 to 500 μg mL^−1^. Specifically, *Salmonella typhi* displayed the highest susceptibility, exhibiting an MIC of 7.8 μg mL^−1^, followed by *Enterobacter cloacae* (MICs of 15.7–31.3 μg mL^−1^). While *Proteus mirabilis* proved to be the most resistant strain, *P. aeruginosa* was uniquely susceptible to the bactericidal action of the oils. Chemical characterization of the floral essential oils revealed that yomogi alcohol, camphor, artemisia alcohol (14.7%), and artemisia acetate (7.3%) constituted the primary components. The observed antibacterial properties are consistent with previous research; for instance, camphor is a recognized antimicrobial agent, and earlier studies on whole-plant EOs have attributed the significant inhibitory effects against *S. aureus* and *C. jejuni* to the presence of both artemisia and yomogi alcohols [17,45].

*Pancratium maritimum* L.

The antimicrobial properties of essential oils and extracts of *P. maritimum* have been extensively evaluated. Tosun et al. (2004) demonstrated that the bulb extract exhibits potent antimycobacterial activity against *Mycobacterium tuberculosis*, reporting a MIC of 100 μg mL^−1^ [65]. In contrast, the antimicrobial efficacy of the aerial parts (stems and leaves) appears to be more limited and highly dependent on the extraction method and chemical composition. Research also indicates that the aerial parts exert only moderate to weak inhibitory effects, although they are rich in alkaloids. Specifically, the alkaloid-enriched fraction has been shown to inhibit *P. aeruginosa* at an MIC of 1125 μg mL^−1^, with considerably higher concentrations required to achieve growth inhibition against *S. aureus, E. coli, and E. faecalis*. Furthermore, recent findings by Melliti et al. (2024) indicated that the methanolic extract of the aerial parts remained inactive against the tested pathogenic strains [19]. This lack of significant antibacterial activity is consistent with reports from previous studies, which suggest that the methanolic constituents may lack the specific bioactive compounds necessary for robust antimicrobial action [70,71,72]. Similarly, the essential oil derived from the aerial parts displayed negligible antimicrobial efficacy, necessitating MIC values exceeding 3250 μg mL^−1^ for all bacterial strains evaluated [52]. These findings collectively suggest that the therapeutic potential of this species is highly localized, with the bioactive compounds, particularly those targeting mycobacterial strains, being concentrated primarily within the bulbous tissues rather than the aerial vegetative structures.

*Salicornia europaea* L.

Although there is growing evidence supporting the antimicrobial efficacy *of S. europaea*, quantitative data such as MIC values are often missing from the literature. Recent research by Limongelli et al. (2025) highlights the effectiveness of polyphenol-rich NADES extracts against a broad spectrum of pathogens, specifically *E. faecalis*, *S. aureus*, *E. coli*, and *K. pneumoniae*, with some preparations showing potency comparable to or exceeding that of levofloxacin [73]. Furthermore, other extraction methods have yielded fewer promising outcomes; Castagna et al. (2022) observed that hydromethanolic extracts were only effective against *B. subtilis* at 1000 μg mL^−1^ [11]. Furthermore, Rahmani et al. (2022) noted that organic leaf and root extracts demonstrated weaker inhibitory activity than the reference standard of ampicillin [55].

**Figure 3:**
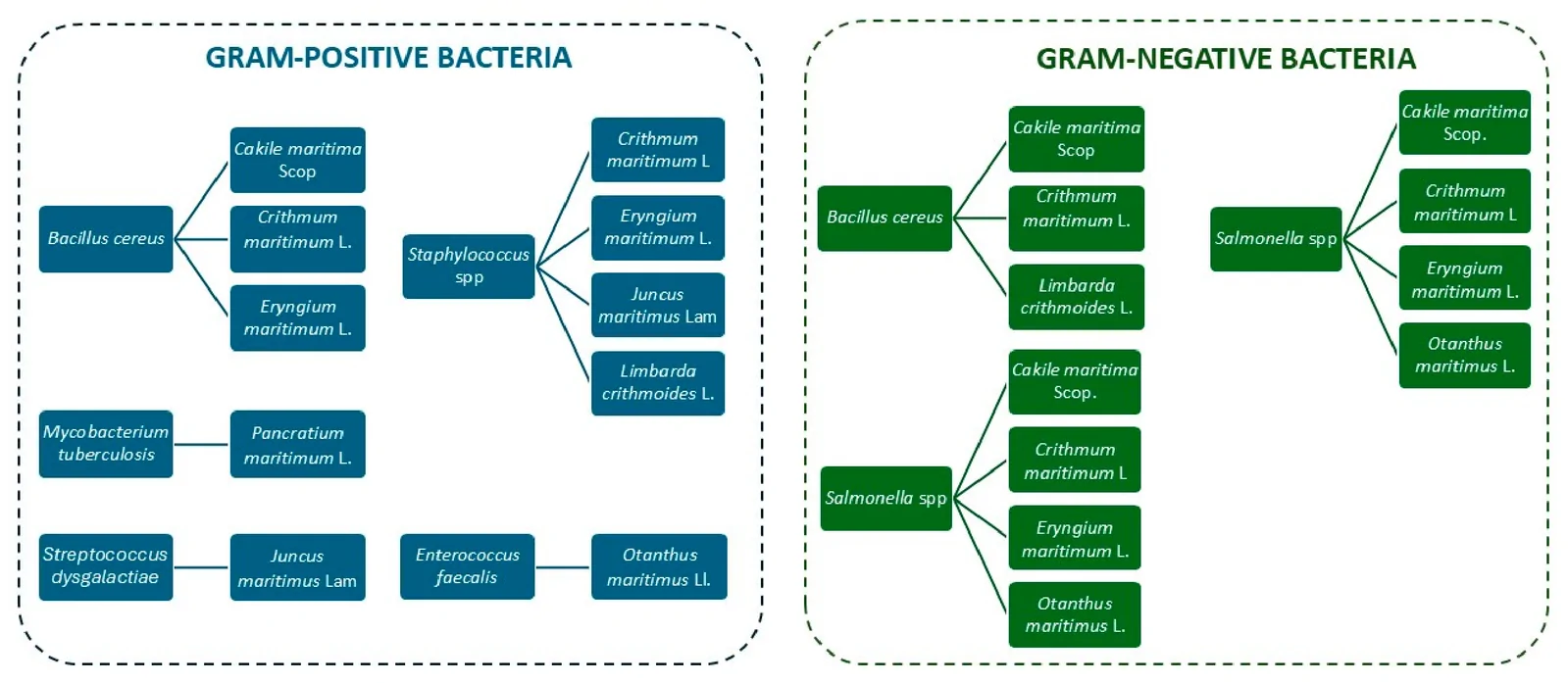
Most active halophyte species against Gram-positive and Gram-negative bacteria. The minimum inhibitory concentration (MIC) cut-off was set at 100 μg mL^−1^.

Although the antimicrobial potential of various halophytes has been extensively documented, identifying the most promising candidates remains challenging due to the lack of standardized reporting units and classification systems across the literature. To establish meaningful guidelines, the MIC values of standard antibiotics typically ranging from 0.01 to 10 μg mL^−1^ are considered [74]. Given that plant extracts and essential oils are complex mixtures, a threshold of MIC <100 μg mL^−1^ was adopted in this study to highlight candidates with significant antimicrobial efficacy. Kuete (2010) also stated that the antimicrobial activity of plant extracts will be classified as significant for MIC values lower than 100 µg mL^−1^ [75]. Based on this criterion, the aerial extracts of *C. maritima*, *C. maritimum*, and *E. maritimum*, the floral extract of *O. maritimus*, and the essential oils derived from the aerial parts of *C. maritimum* are identified as the most promising halophyte materials for antimicrobial applications. Furthermore, the efficacy of these halophytes is categorized by bacterial genus and species. Figure 3 provides a summary of the most active halophytes against frequently studied bacteria, utilizing a cut-off value of 100 μg mL^−1^.

## 4. Antifungal Activity of Halophytes

The potential of halophytic plants to address the challenges posed by both clinical and agricultural fungal pathogens is increasingly recognized as a paramount priority of current research. These halophytes, which thrive in challenging saline environments, have demonstrated a broad spectrum of inhibitory activity against various Candida species, as well as several economically significant phytopathogens. Research indicates that the effectiveness of these plants is frequently dependent on the specific tissue utilized, such as flowers, leaves, or schizocarps and the extraction method employed. While some species exhibit potent activity against Candida strains and other yeasts, others show significant promise in managing filamentous fungi and agricultural threats like Fusarium, Alternaria, and Phytophthora. Table 2 summarizes the antifungal activity of various halophyte extracts and essential oils against specific fungal strains.

*Crithmum maritimum* L.

The essential oils and extracts of this halophyte exhibit significant antifungal activity. Studies have demonstrated the efficacy of its essential oils against *Candida albicans*, *Candida guilliermondii*, and various dermatophytes [76], alongside promising activity against *Cryptococcus neoformans* (MIC = 260 μg mL^−1^). This bioactivity is largely attributed to sabinene, a major monoterpene known to suppress *C. albicans* virulence factors, such as germ tube and biofilm formation [77].

Further evaluation of apolar extracts from *C. maritimum* tissues (leaves, flowers, and schizocarps) against multiple fungal strains confirmed strong inhibition of *Candida* species. Specifically, *C. albicans* were inhibited at an MIC of 75 μg mL^−1^ and a minimum fungicidal concentration of 150 μg mL^−1^. Notably, extracts from reproductive tissues (flowers and schizocarps) generally outperformed foliar extracts. Furthermore, efficacy was solvent-dependent; petroleum ether extracts from schizocarps were most effective against *C. albicans* and *C. parapsilosis*, whereas acetonic flower extracts demonstrated superior inhibition of *C. albicans* [57].

*Eryngium maritimum* L.

While the antimicrobial properties of E. *maritimum* have been extensively documented, there is a notable paucity of research regarding its antifungal potential. Existing studies indicate that polar extracts derived from the leaves and roots exhibit promising inhibitory activity against *Candida glabrata*, alongside moderate to weak efficacy against *C. albicans*. Furthermore, research has highlighted significant inhibitory effects against *Aspergillus niger,* a ubiquitous and destructive fungal pathogen in horticultural systems [78]. Conversely, evaluations of the plant’s essential oils suggest that high concentrations are required to achieve meaningful fungal growth inhibition, thereby limiting their practical utility as therapeutic or agricultural antifungal agents [12,67].

*Limbarda (Inula) crithmoides* L.

The antifungal capacity of this halophyte against three economically significant agricultural pathogens was reported. Growth inhibition of *Phytophthora cryptogea* and *Alternaria solani* was achieved using both hexanic and methanolic aerial extracts. The methanolic extract displayed a potency comparable to nystatin at a concentration of 400 μg mL^−1^ [79]. Furthermore, MIC measurements indicated that these extracts are also effective against *Fusarium oxysporum*, corroborating earlier studies that highlighted the antifungal properties of methanolic flower extracts against the same species [80].

*Otanthus maritimus* L.

The essential oil derived from the flowers of *O. maritimus* was characterized by a high proportion of oxygenated monoterpenes, which are known for their broad-spectrum biological activities. The antifungal potential of these oils was evaluated against several phytopathogenic fungi, revealing differential sensitivity among the tested strains. *Rhizoctonia solani* exhibited the highest susceptibility to the essential oils, with a MIC of 62.5 μg mL^−1^, suggesting potent fungistatic activity. In contrast, *C. albicans* and *Botrytis cinerea* demonstrated lower sensitivity, recording identical MIC values of 125 μg mL^−1^ [17]. These findings indicate that the oxygenated monoterpene-rich profile of *O. maritimus* oil possesses significant efficacy against filamentous phytopathogens, highlighting its potential as a natural alternative for crop protection and fungal growth control.

*Pancratium maritimum* L.

Significant antifungal activity has been documented for the essential oil of *P. maritimum* L. against various *Candida* species. Specifically, its oils effectively inhibit the growth of *C. albicans* and *C. parapsilosis* at concentrations below 1000 μg mL^−1^, whereas a higher MIC value of 13,000 μg mL^−1^ is required for *C. glabrata* [52]. This antifungal activity may be attributed to a synergistic effect of diverse phytoconstituents, including 3,5,5-trimethylcyclohex-3-en-1-one, a compound recognized for its intrinsic antifungal properties, alongside phytol, fenchone, and limonene, all of which have independently demonstrated significant efficacy against *Candida* strains. Earlier investigations by Sür-Altiner et al. (1999) corroborate these findings, noting that while the methanolic extract of *P. maritimum* bulbs lacked antibacterial activity, it exhibited broad-spectrum efficacy against yeast species. Notably, at a concentration of 60 μg per disc, the extract displayed antifungal activity comparable to the clinical reference drug miconazole against *Candida pseudotropicalis*, *C. krusei*, *C. tropicalis*, and *C. guillermondii* [81]. Furthermore, although the alkaloid-enriched apolar organic extracts from flowers and fruits exhibit a relatively weaker inhibitory effect on *Candida* species, they retain significant potential for the control of plant fungal pathogens, including *Rhizopus microsporus* and *Aspergillus fumigatus* [72].

The present review synthesizes current research regarding the antifungal efficacy of these Mediterranean halophytes, focusing mainly on the utilization of essential oils and apolar extracts for the control of fungal pathogens with important clinical and agricultural implications. Candida species represent the most extensively studied clinical pathogens, with *C. maritimum*, *E. maritimum*, and *P. maritimum* demonstrating significant potential to inhibit the growth of various Candida strains. Beyond their clinical relevance, these halophytes exert antifungal properties against a broad spectrum of agricultural pathogens that are responsible for extensive post-harvest losses in fruits, vegetables, and cereals, most notably *F. oxysporum*, *A. solani*, *P. cryptogea*, and *R. solani*. The antimicrobial capacity of these halophytes is frequently linked to their bioactive contents, such as oxygenated monoterpenes and alkaloids, which exhibit differential efficacy based on the plant tissue source and the specific extraction solvent employed. This study underscores the potential of these resilient Mediterranean species to serve as natural alternatives to synthetic fungicides in both pharmaceutical and agrifood systems.

**Table 2:** Antifungal activity of halophyte-derived extracts and essential oils, detailing the plant parts, extraction methods, and respective minimum inhibitory concentration (MIC) values as determined by broth microdilution assays.

Halophyte	Plant Part	Type of Extract	Fungi	MIC (μg mL^−1^)	Reference
*Crithmum maritimum*	Leaves Schizocarps Flowers	Acetone Essential oils Ethanol Acetone Petroleum ether Acetone Petroleum ether	*Candida albicans* *Candida guillermondii* *Candida albicans* *Candida albicans* *Candida parapsilosis* *Komagataella phaffii* *Saccharomyces cerevisiae* *Candida albicans* *Candida parapsilosis* *Komagataella phaffii* *Saccharomyces cerevisiae* *Candida albicans* *Candida parapsilosis* *Candida albicans* *Candida parapsilosis* *Komagataella phaffii*	600 640 600 75 300 150 300 75 150 300 300 75 300 150 300 150	[57] [76] [57]
*Eryngium maritimum*	Leaves Roots	70% ethanol 70% ethanol	*Candida glabrata* *Aspergillus niger* *Candida glabrata* *Aspergillus niger*	700 700 400 700	[78]
*Limbarda crithmoides*	Aerial parts	Methanol Hexane	*Fusarium oxysporum* *Alternaria solani* *Phytophthora cryptogea* *Fusarium oxysporum* *Alternaria solani* *Phytophthora cryptogea*	250 250 250 250 250 250	[79]
*Otanthus maritimus*	Flowers Aerial parts	Essential oil Essential oil	*Rhizoctonia solani* *Botrytis cinerea* *Candida albicans* *Cryptococcus neoformans*	62.5 123 125 ~640	[17] [82]
*Pancratium maritimum*	Aerial parts Flowers Fruits	Essential oil Petroleum ether Petroleum ether	*Candida albicans* *Candida parapsilosis* *Aspergillus fumigatus* *Rhizopus microspores* *Rhizopus microspores*	810 405 490 60 240	[52] [72]

## 5. The Potential of Halophytes as Natural Antimicrobial Agents in Agrifood Sector

Natural preservative in foods

The increasing consumer preference for clean-label, plant-derived alternatives has shifted research focus toward halophytes as sustainable, high-potential sources of bioactive compounds for food safety and shelf-life extension. Rich in secondary metabolites such as polyphenols, essential oils, saponins, alkaloids, phenathrenoids, and sulfur compounds, halophytes offer promising in vitro antimicrobial properties capable of effectively inhibiting common foodborne pathogens, including *E. coli*, *Salmonella* spp., *L. monocytogenes*, and *Candida* spp. Research highlights the significant inhibitory potential of halophyte extracts against prominent foodborne pathogens. For instance, studies have demonstrated the efficacy of *C. maritimum* (sea fennel) in suppressing *E. coli*, a primary contaminant in leafy greens, meat, and dairy matrices [56,58,59]. Furthermore, apolar extracts and essential oils derived from *C. maritima*, *C. maritimum*, *E. maritimum*, and *O. maritimus* have been identified as inhibitors of various *Salmonella* species in poultry and seafood sectors [19,59]. Beyond antibacterial activity, the antifungal potential of halophytes is particularly relevant for the preservation of high-sugar or acidic products, such as fruit juices, jams, and dairy, where *Candida* species often drive spoilage. Recent studies report the utility of *C. maritimum, E. maritimum, O. maritimus, and P. maritimum* as natural antifungal agents [52,57,78].

Despite this promise, the industrial application of these extracts faces significant challenges. Direct incorporation into the food matrix is frequently inhibited by poor water solubility, limited chemical stability, and susceptibility to oxidative or thermal degradation during processing. Furthermore, the high concentrations often required for efficacy can trigger undesirable organoleptic shifts, such as pronounced bitterness or astringency derived from high polyphenol contents [83]. To circumvent these limitations, current research advocates for the integration of halophyte-derived bioactives into active packaging systems. Strategies include the development of antimicrobial films, the deposition of bioactive coatings, and the surface grafting of phenolic compounds onto food-contact materials. These approaches facilitate a controlled, sustained release of active agents, effectively mitigating cross-contamination and biofilm formation on contact surfaces while preserving the sensory profile of the food product [84]. By decoupling the antimicrobial agent from the food matrix, active packaging offers an alternative pathway for enhancing microbial safety, though translating these systems to commercial food preservation requires overcoming real-world matrix stability challenges.

Management of plant-pathogenic fungi

The utilization of halophytic extracts to control plant pathogenic fungi represents a highly promising alternative to conventional synthetic fungicides. These extracts exhibit notable antifungal properties due to the diverse secondary metabolites, such as alkaloids, flavonoids, phenolic compounds, and essential oils [85]. They actively suppress fungal growth by disrupting cell membrane integrity, inhibiting vital enzyme activities, and preventing spore germination. Furthermore, because these botanical formulations are biodegradable and possess complex, multi-site modes of action, they significantly reduce the possibility of pathogens developing resistance while minimizing environmental toxicity and risks to non-target organisms [86]. Integrating these eco-friendly extracts into integrated pest management strategies not only protects crop yields but also supports the global shift toward greener agricultural practices. The reliance on conventional synthetic fungicides for the management of pre- and post-harvest phytopathogens has necessitated the exploration of sustainable alternatives due to concerns regarding environmental toxicity, pathogen resistance, and chemical residues in the food chain [87]. Recent studies underscore the bioactivity of several halophytic species, namely *L. crithmoides*, *P. maritimum*, and *O. maritimus*, as potential sources of antifungal compounds. These species have demonstrated in vitro efficacy against a broad spectrum of economically devastating agricultural pathogens, including *F. oxysporum*, *A. solani*, *P. cryptogea*, *R. solani*, *B. cinerea*, and *R. microsporus* [17,71,79]. Furthermore, preliminary investigations suggest that extracts derived from *P. maritimum* exhibit antifungal activity against *A. fumigatus* and *A. niger*. As primary contaminants of stored grains, fruits, and vegetables, these *Aspergillus* species not only contribute to significant post-harvest quantitative losses through rapid spoilage but also pose a critical food safety threat due to the accumulation of hazardous mycotoxins [74,78].

Despite these encouraging in vitro results, a substantial knowledge gap remains. The efficacy of these extracts and essential oils has not been evaluated under field trials or within commercial storage environments. Moreover, the environmental stability and residual persistence of these halophytic extracts under fluctuating field conditions remain underestimated. Consequently, further systematic research is required to elucidate the stability and performance of these botanical agents as viable, field-applied, or post-harvest integrated fungi management tools.

Formulation of new prebiotics

The shifting of nutritional science, driven by a growing interest in gut health, has increasingly focused on the identification of “next-generation” prebiotics, phytoconstituents capable of selectively modulating the gut microbiota to promote host health. Accumulating evidence indicates that various plant-derived bioactive compounds can survive upper gastrointestinal digestion and engage in bidirectional interactions with the colonic microbial ecosystem, a process in which halophytes represent a vast, untapped reservoir [88]. By synthesizing diverse bioactive profiles, including complex polysaccharides, proteins, and antimicrobial secondary metabolites, halophytes offer a promising candidate source for exploring prebiotic substrates, although extensive in vivo and clinical evaluations are required to confirm health benefits. Studies on *C. maritimum* (sea fennel) have demonstrated that its leaf extracts significantly accelerate the growth kinetics of *Lactobacilli* and provide a matrix for the delivery of probiotic strains such as *Lactiplantibacillus plantarum* IMC 509. With a nutrient profile characterized by high carbohydrate content (66.5%), sea fennel effectively supports microbial survival and metabolic activity in preliminary trials, highlighting its potential for functional food development [57,89]. Given these preliminary successes, the systematic evaluation of halophytic diversity is poised to attract significant scientific and industrial interest, offering a novel strategy for the development of functional foods and therapeutic interventions for diet-related pathologies.

Ruminant well-being

The traditional utilization of halophytes in practices, such as *Atriplex* spp. and *Plantago* spp., highlights their suitability for animal maintenance and productivity [90]. Furthermore, halophytes are recognized for their abundance of bioactive compounds, contributing to improved animal health, well-being, and improved quality of the animal products for human health [90,91]. Plant extracts and essential oils are utilized to control many bacterial and fungal diseases affecting animals, which cause important economic losses in livestock, and modulate microbial activity in the rumen. According to Table 1, the halophyte extracts and essential oils exhibit antimicrobial potential against several bacterial pathogens responsible for various ruminant diseases. Specifically, preliminary in vitro data suggest extracts from C. *maritimum, E. maritimum, J. maritimus, L. crithmoides,* and *O. maritimus* show promise in addressing mastitis, as they demonstrate inhibitory activity against causative agents such as *S. aureus, S. agalactiae, S. dysgalactiae*, and *E. faecalis* [17,57,61,63]. Furthermore, extracts derived from *C. maritima*, *C. maritimum, E. maritimum*, and *O. maritimus* could be utilized to combat enteric infections and diarrhoea, given their efficacy against pathogens including *B. cereus*, *Salmonella* spp., *Proteus* spp., and *Enterobacter* spp. [17,56,57]. Additionally, these extracts may play a role in managing respiratory infections by targeting bacteria such as *Bacillus* species, which are frequently implicated in complex respiratory conditions. The therapeutic potential of *P. maritimum* against *M. tuberculosis* has been reported [65]. Such efforts are particularly significant in light of the “reverse zoonosis” transmission of *M. tuberculosis* from humans to livestock, which necessitates a One Health approach to disease control and management.

Several halophyte extracts also exhibit antifungal potential against various fungal strains, which may have implications for managing mycotic diseases in livestock. Extracts from *C. maritimum*, *E. maritimum*, and *P. maritimum* have demonstrated inhibitory activity against *Candida* species and *Aspergillus* strains, which are relevant to superficial and systemic fungal infections in animals [19,57,76,78]. Furthermore, *O. maritimus* has been evaluated for its effectiveness against *Cryptococcus neoformans*, which is primarily associated with mastitis [87]. These findings highlight the potential for incorporating halophyte-derived compounds into strategies for controlling fungal pathogens in livestock environments.

## 6. Method

A targeted search of electronic databases, including PubMed, Web of Science, Scopus, and Google Scholar, was conducted to identify relevant studies published between 2000 and 2026 using the Boolean search string: (‘antimicrobial’ OR ‘antifungal’ OR ‘antibacterial’) AND (‘activity’ OR ‘effect’) AND ‘halophyte’. The search was restricted to original, peer-reviewed articles published in English. Articles were selected based on the following inclusion criteria: (i) studies focusing on extracts or essential oils from halophytes distributed across at least five countries within the Mediterranean basin; (ii) reporting of minimum inhibitory concentration (MIC) values below 2000 $\mu {g\;\mathrm{mL}}^{-1}$; (iii) demonstrated antimicrobial activity against common foodborne bacteria and fungi; (iv) a fully characterized phytochemical composition of the extracts and/or essential oils; and (v) presentation of original primary data. Studies were excluded if they contained incomplete data, comprised secondary or gray literature, or involved duplicate datasets. This selection strategy identified nine halophyte species that fulfilled all requirements regarding widespread Mediterranean distribution, established antimicrobial potency relevant to agricultural microbiology, and well-defined phytochemical profiles (Figure 1). Notably, this narrative describes a topic-guided literature search methodology rather than an exhaustive systematic review adhering strictly to PRISMA 2020 guidelines.

## 7. Conclusions & Future Perspectives

The Mediterranean basin possesses a rich diversity of halophytic flora, which has evolved unique metabolic pathways to thrive under high salinity, drought, and ultraviolet radiation. These physiological adaptations facilitate the accumulation of high-value bioactive secondary metabolites, which exhibit significant antimicrobial and antifungal potential. This review identifies nine Mediterranean halophytes whose extracts and essential oils demonstrate remarkable inhibitory effects against clinical, foodborne, and agricultural pathogens, frequently exhibiting MIC values comparable to or lower than established antibiotic substances. Although antimicrobial evaluations are primarily restricted to lab-scale studies, these halophytes offer new, promising approaches for food preservation, integrated pest management for crop protection, and the development of functional prebiotic additives. Furthermore, their application in ruminant nutrition offers a promising approach to mitigating enteric infections, mastitis, and respiratory diseases, thereby improving overall livestock welfare and production efficiency. Despite these promising preliminary findings, several critical technological and translational barriers must be resolved to bridge the gap between bench-scale bioassays and commercial solutions. Crucially, while in vitro potency has been demonstrated, the performance of halophytic extracts and essential oils under complex matrices, such as agricultural field conditions, commercial food storage environments, and the dynamic ruminant gastrointestinal lumen, remains almost unvalidated. Furthermore, the direct application of crude extracts and essential oils into food or feed matrices is frequently constrained by poor aqueous solubility, susceptibility to thermal degradation, and undesirable organoleptic attributes. Overcoming these limitations necessitates advanced formulation strategies, such as microencapsulation and active packaging, to preserve bioactivity and ensure controlled kinetics. Finally, comprehensive toxicological profiling and safety evaluations are essential to establish reliable exposure thresholds prior to their commercialization.

## Figures and Tables

**Table 1:** Antibacterial activity of halophyte-derived extracts and essential oils, detailing the plant parts, extraction methods, and respective minimum inhibitory concentration (MIC) values as determined by broth microdilution assays.

Halophyte	Plant Part	Type of Extract	Bacteria	MIC (μg mL^−1^)	Reference
*Cakile maritima L.*	Leaves Seeds	Methanol Chloroform Ethanol	*Pseudomonas marginalis* *Pseudomonas aeruginosa* *Salmonella arizonae* *Salmonella montevideo* *Pseudomonas marginalis* *Pseudomonas aeruginosa* *Bacillus cereus* *Acinetobacter baumannii* *Escherichia coli* *Enterococcus faecalis* *Klebsiella pneumoniae* *Pseudomonas aeruginosa* *Staphylococcus aureus*	2 1 10 100 100 1 100 150 75 150 150 75 150	[56] [20]
*Crithmum maritimum*	Flowers Schizocarps Leaves Aerial parts	Ethanol Acetone Ethanol Acetone Petroleum ether 80% ethanol Methanol Petroleum ether Hydro-distillation Chloroform Commercial essential oils	*Staphylococcus aureus* *Staphylococcus epidermis* *Staphylococcus aureus* *Staphylococcus aureus* *Staphylococcus aureus* *Staphylococcus aureus* *Staphylococcus epidermis* *Escherichia coli* *Salmonella typhimurium* *Enterobacter aerogenes* *Enterococcus faecalis* *Staphylococcus aureus* *Salmonella arizonae* *Pseudomonas marginalis* *Pseudomonas aeruginosa* *Pseudomonas fluorescens* *Staphylococcus aureus* *Staphylococcus epidermis* *Staphylococcus aureus* *MRS-Staphylococcus aureus* *Listeria innucia* *Escherichia coli* *Salmonella arizonae* *Pseudomonas aeruginosa* *Pseudomonas fluorescens* *Escherichia coli* *Bacillus cereus* *Campylobacter jejuni*	300 300 75 300 75 75 150 250 250 250 250 250 1 100 1 1 75 150 ~110 ~110 ~110 ~300 1 100 100 100 10 ~300	[20] [57] [56] [58] [59] [60]
*Eryngium maritimum*	Leaves Roots Aerial parts	Methanol Chloroform Dichloromethane Petroleum ether Dichloromethane Petroleum ether	*Pseudomonas aeruginosa* *Pseudomonas fluorescens* *Staphylococcus aureus* *Salmonella arizonae* *Salmonella montevideo* *Pseudomonas aeruginosa* *Pseudomonas fluorescens* *Bacillus cereus* *Stenotrophomonas maltophilia* *Staphylococcus lugdunensis* *Stenotrophomonas maltophilia* *Yersinia pseudotuberculosis* *Staphylococcus lugdunensis* *Streptococcus dysgalactiae* *Stenotrophomonas maltophilia* *Staphylococcus epidermidis* *Staphylococcus lugdunensis* *Staphylococcus warneri* *Streptococcus dysgalactiae* *Acinetobacter baumannii* *Stenotrophomonas maltophilia* *Yersinia pseudotuberculosis* *Staphylococcus epidermidis* *Staphylococcus lugdunensis*	1 1 10 100 100 2 2 1 78 625 156 625 156 625 156 625 156 625 625 312 156 625 625 312	[56] [61]
*Juncus acutus*	Whole plant	Dichloromethane	*MRS-Staphylococcus aureus*	156	[62]
*Juncus maritimus*	Rhizomes Whole plant	Water/Dichloromethane/ethyl acetate Dichloromethane	*Staphylococcus lugdunensis* *Streptococcus agalactiae* *Streptococcus dysgalactiae* *MRS-Staphylococcus aureus*	625 625 78 78	[63] [62]
*Limbarda crithmoides*	Aerial parts	Hydro-distillation	*Escherichia coli* *Staphylococcus aureus*	3.12 6.25	[64]
*Otanthus maritimus*	Flower	Hydro-distillation	*Staphylococcus aureus Enterococcus faecalis* *Proteus vulgaris* *Proteus mirabilis* *Salmonella typhi* *Enterobacter cloacae* *Enterobacter aerogenes* *Pseudomonas aeruginosa*	125–250 62.5–125 31.3–62.5 250–500 7.8 15.7–31.3 125–250 31.3–62.5	[17]
*Pancratium maritimum*	Bulbs	70% ethanol	*Mycobacterium tuberculosis*	100	[65]
*Salicornia europaea L.*	Aerial parts	80% methanol	*Bacillus subtilis*	1000	[11]

## Data Availability

No new data were created or analyzed in this study. Data sharing is not applicable to this article.

## References

[1] Castañeda-Loaiza V., Oliveira M., Santos T., Schüler L., Lima A.R., Gama F., Salazar M., Neng N.R., Nogueira J.M.F., Varela J. (2020). Wild vs. Cultivated Halophytes: Nutritional and Functional Differences. Food Chem..

[2] Wungrampha S., Rawat N., Singla-Pareek S.L., Pareek A., Grigore M.-N. (2021). Survival Strategies in Halophytes: Adaptation and Regulation. Handbook of Halophytes: From Molecules to Ecosystems Towards Biosaline Agriculture.

[3] Yuan F., Xu Y., Leng B., Wang B. (2019). Beneficial Effects of Salt on Halophyte Growth: Morphology, Cells, and Genes. Open Life Sci..

[4] Hussain S., Khalid M.F., Sohail M., Anjum M.A., Ejaz S., Nafees M., Zakir I., Ahmad M., Ali S., Ahmad S., Hasanuzzaman M. (2021). Role of Transporters in Accumulating Salt Ions by Halophytes. Approaches to the Remediation of Inorganic Pollutants.

[5] Petropoulos S.A., Karkanis A., Martins N., Ferreira I.C.F.R. (2018). Halophytic Herbs of the Mediterranean Basin: An Alternative Approach to Health. Food Chem. Toxicol..

[6] Lopes M., Sanches-Silva A., Castilho M., Cavaleiro C., Ramos F. (2023). Halophytes as Source of Bioactive Phenolic Compounds and Their Potential Applications. Crit. Rev. Food Sci. Nutr..

[7] Solomou A.D. (2025). Mediterranean Basin. Terrestrial Biomes: Global Biome Conservation and Global Warming Impacts on Ecology and Biodiversity.

[8] Mastrocicco M., Colombani N. (2021). The Issue of Groundwater Salinization in Coastal Areas of the Mediterranean Region: A Review. Water.

[9] Karnwal A., Malik T. (2024). Exploring the Untapped Potential of Naturally Occurring Antimicrobial Compounds: Novel Advancements in Food Preservation for Enhanced Safety and Sustainability. Front. Sustain. Food Syst..

[10] Salam M.A., Al-Amin M.Y., Salam M.T., Pawar J.S., Akhter N., Rabaan A.A., Alqumber M.A.A. (2023). Antimicrobial Resistance: A Growing Serious Threat for Global Public Health. Healthcare.

[11] Castagna A., Mariottini G., Gabriele M., Longo V., Souid A., Dauvergne X., Magné C., Foggi G., Conte G., Santin M. (2022). Nutritional Composition and Bioactivity of *Salicornia Europaea* L. Plants Grown in Monoculture or Intercropped with Tomato Plants in Salt-Affected Soils. Horticulturae.

[12] Majdoub S., El Mokni R., Debbabi H., Chaabane-Banaoues R., AL-Hmadi H.B., Chaieb I., Piras A., Babba H., Porcedda S., Hammami S. (2025). Phytochemical Composition, Antifungal and Insecticidal Properties of the Essential Oils of Three Species of the Genus *Eryngium Tourn*. Ex L. (Apiaceae, Saniculoideae) Growing Natively in Tunisia. Chem. Biodivers..

[13] Dzhoglova V., Ivanova S., Shishmanova-Doseva M., Saracheva K. (2025). *Crithmum Maritimum* L.: Phytochemical Profile, Biological Activities, and Therapeutic Potential. Molecules.

[14] Bús C., Tóth B., Stefkó D., Hohmann J., Vasas A. (2018). Family Juncaceae: Promising Source of Biologically Active Natural Phenanthrenes. Phytochem. Rev..

[15] Brzović P., Radman S., Mekinić I.G. (2025). Phenolic Antioxidants in the Adriatic Halophyte *Limbarda Crithmoides*: Variation Across Phenological Stages. Foods.

[16] Jallali I., Snoussi M., Zaouali Y., Hannachi H., Ksouri R., Ahmed H. (2026). Ben Statistical Investigation of *Limbarda Crithmoïdes* (L.) Dumort Subsp. Longifolia Essential Oils Phytochemistry Variation and Related Biological Activities According to the Plant Organ and the Geographic Origin. J. Essent. Oil Bear. Plants.

[17] Basile A., Rigano D., Sorbo S., Conte B., Rosselli S., Bruno M., Senatore F. (2013). Antibacterial and Antifungal Activities of *Otanthus Maritimus* (L.) Hoffmanns. & Link Essential Oil from Sicily. Nat. Prod. Res..

[18] Christodoulopoulou L., Tsoukatou M., Tziveleka L.A., Vagias C., Petrakis P.V., Roussis V. (2005). Piperidinyl Amides with Insecticidal Activity from the Maritime Plant *Otanthus Maritimus*. J. Agric. Food Chem..

[19] Melliti M., Musa E.M., Skhiri F., Edziri H. (2024). HPLC Analysis, Antioxidant, Antimicrobialactivities of Alcaloids and Methanolic Extract of *Pancratium Maritimum* Growing in Djerba. Arch. Pharm. Pract..

[20] Fuochi V., Barbagallo I., Distefano A., Puglisi F., Palmeri R., Di Rosa M., Giallongo C., Longhitano L., Fontana P., Sferrazzo G. (2019). Biological properties of *Cakile maritima* Scop. (Brassicaceae) extracts. Eur. Rev. Med. Pharmacol. Sci..

[21] Iranshahi M. (2012). A Review of Volatile Sulfur-Containing Compounds from Terrestrial Plants: Biosynthesis, Distribution and Analytical Methods. J. Essent. Oil Res..

[22] Ashim A., Ismaiel L., Fanesi B., Nartea A., Maoloni A., Orhotohwo O.L., Darko H.S.O., Lucci P., Aquilanti L., Pacetti D. (2025). Food-Grade Polar Extracts from Sea Fennel (*Crithmum Maritimum* L.) By-Products: Unlocking Potential for the Food Industry. Foods.

[23] Pal S., Panda A., Bhowmick B., Bera K. (2025). *Crithmum Maritimum*: Phytoconstituents Composition, and Potential Anticancer Activity in Gastrointestinal Cancer Mechanisms and Prospects. Harmacol. Res.—Mod. Chin. Med..

[24] Generalić Mekinić I., Politeo O., Ljubenkov I., Mastelić L., Popović M., Veršić Bratinčević M., Šimat V., Radman S., Skroza D., Ninčević Runjić T. (2024). The Alphabet of Sea Fennel: Comprehensive Phytochemical Characterisation of Croatian Populations of *Crithmum Maritimum* L.. Food Chem. X.

[25] Politeo O., Ćurlin P., Brzović P., Auzende K., Magné C., Generalić Mekinić I. (2024). Volatiles from French and Croatian Sea Fennel Ecotypes: Chemical Profiles and the Antioxidant, Antimicrobial and Antiageing Activity of Essential Oils and Hydrolates. Foods.

[26] Dzhoglova V., Ivanov K., Benbassat N., Georgieva-Dimova Y., Ardasheva R., Karcheva-Bahchevanska D., Ivanova S. (2024). *Crithmum Maritimum* L.—Study on the Histochemical Localization of Essential Oil. Plants.

[27] Zafeiropoulou V., Tomou E.M., Douros A., Skaltsa H. (2021). The Effect of Successive Harvesting on The Volatile Constituents of Two Essential Oils of Cultivated Populations of Sea Fennel (*Crithmum Maritimum* L.) in Greece. J. Essent. Oil-Bear. Plants.

[28] Nakurte I., Berga M., Mežaka I. (2023). Phytochemical Diversity Comparison in Leaves and Roots of Wild and Micropropagated Latvian Sea Holly (*Eryngium Maritimum* L.). Molecules.

[29] Kikowska M., Chanaj-Kaczmarek J., Derda M., Budzianowska A., Thiem B., Ekiert H., Szopa A. (2022). The Evaluation of Phenolic Acids and Flavonoids Content and Antiprotozoal Activity of Eryngium Species Biomass Produced by Biotechnological Methods. Molecules.

[30] Erdem S.A., Nabavi S.F., Orhan I.E., Daglia M., Izadi M., Nabavi S.M. (2015). Blessings in Disguise: A Review of Phytochemical Composition and Antimicrobial Activity of Plants Belonging to the Genus Eryngium. DARU J. Pharm. Scie..

[31] Maggio A., Bruno M., Formisano C., Rigano D., Senatore F. (2013). Chemical Composition of the Essential Oils of Three Species of Apiaceae Growing Wild in Sicily: *Bonannia Graeca, Eryngium Maritimum and Opopanax Chironium*. Nat. Prod. Comm..

[32] Dellagreca M., Isidori M., Lavorgna M., Monaco P., Previtera L., Zarrelli A. (2004). Bioactivity of Phenanthrenes from *Juncus Acutus* on *Selenastrum Capricornutum*. J. Chem. Ecol..

[33] DellaGreca M., Fiorentino A., Isidori M., Previtera L., Temussi F., Zarrelli A. (2003). Benzocoumarins from the Rhizomes of *Juncus Acutus*. Tetrahedron.

[34] Dellagreca M., Previtera L., Zarrelli A. (2005). Dimeric Phenanthrenoids from Juncus Acutus. Nat. Prod. Res..

[35] Awaad A.S. (2006). Phenolic Glycosides of *Juncus Acutus* and Its Anti-Eczematic Activity. Chem. Nat. Compd..

[36] Hammouti Y., Elbouzidi A., Taibi M., Bellaouchi R., Loukili E.H., Bouhrim M., Noman O.M., Mothana R.A., Ibrahim M.N., Asehraou A. (2023). Screening of Phytochemical, Antimicrobial, and Antioxidant Properties *of Juncus Acutus* from Northeastern Morocco. Life.

[37] Basas-Jaumandreu J., de las Heras F.X.C. (2025). Chemical Investigation of Stem and Fruit Lipophilic Extracts from *Juncus Maritimus*. Biochem. Syst. Ecol..

[38] Kúsz N., Stefkó D., Barta A., Kincses A., Szemeréd N., Spengler G., Hohmann J., Vasas A. (2021). Juncaceae Species as Promising Sources of Phenanthrenes: Antiproliferative Compounds from *Juncus Maritimus* Lam. Molecules.

[39] Zeroual S., Daoud I., Gaouaoui R., Kherachi R. (2025). Polyphenolic Profile and In Vitro, In Silico Study of Stem Extracts from *J. Maritimus* (Juncaceae) Harvested from Eastern Algeria as Potential Anti-Inflammatory and Antioxidant Agents. Chem. Biodivers..

[40] Jallali I., Ben Jemaa M., Medini F., Waffo-Téguo P., Mérillon J.-M., Ben Ahmed H., Ksouri R. (2025). Isolation of the Major Phenolic of *Limbarda Crithmoïdes* L. and Evaluation of Its Contribution in the Phytochemical Variability and the Biological Activities among Localities and Organs. Biochem. Syst. Ecol..

[41] Adorisio S., Giamperi L., Ada Bucchini A.E., Delfino D.V., Marcotullio M.C. (2020). Bioassay-Guided Isolation of Antiproliferative Compounds from *Limbarda Crithmoides* (L.) Dumort. Molecules.

[42] D’Agostino G., Badalamenti N., Franco P., Bruno M., Gallo G. (2022). The Chemical Composition of the Flowers Essential Oil of *Inula Crithmoides* (Asteraceae) Growing in Aeolian Islands, Sicily (Italy) and Its Biocide Properties on Microorganisms Affecting Historical Art Crafts. Nat. Prod. Res..

[43] Fontana G., Bruno M., Senatore F., Formisano C. (2014). Volatile Constituents of Aerial Parts of Two Mediterranean Species of Inula: *Inula Crithmoides* L. and *I. Verbascifolia* (Willd.) Hausskn. (Asteraceae). Nat. Prod. Res..

[44] Parracho T., Cruz P.F., Peralta C.C., Silva C.G., Campos M.J., Neves M., Cordeiro R., Trindade D., Moura C., Almeida Z.L. (2025). Preserving and Hydrogel-Matrixing the Bioactive Properties of Aromatic Medicinal Halophytic Herbs from the Coastline of the Iberian Peninsula. Food Hydrocoll. Health.

[45] Muselli A., Rossi P.-G., Desjobert J.-M., Bernardini A.-F., Berti L., Costa J. (2007). Chemical Composition and Antibacterial Activity of *Otanthus Maritimus* (L.) Hoffmanns. & Link Essential Oils from Corsica. Flavour. Fragr. J..

[46] Tsoukatou M., Vagias C., Harvala C., Roussis V. (2000). Essential Oil and Headspace Analysis of the Maritime *Bombycilaena Erecta* and *Otanthus Maritimus* Species Growing Wild in Greece. J. Essent. Oil Res..

[47] Benabdelkader T., Neghliz H., Jullien F. (2025). Essential Oil of *Otanthus Maritimus* (L.) Hoffmanns. & Link From Algeria With High Content in (α/β)-Isocomene and α-Epi-Bisabolol. Chem. Biodivers..

[48] Zhou H., Ren J., Li Z. (2017). Antibacterial Activity and Mechanism of Pinoresinol from *Cinnamomum Camphora* Leaves against Food-Related Bacteria. Food Control.

[49] Mahendra Kumar C., Singh S.A. (2015). Bioactive Lignans from Sesame (*Sesamum Indicum* L.): Evaluation of Their Antioxidant and Antibacterial Effects for Food Applications. J. Food Sci. Technol..

[50] Le N.-T.-H., De Jonghe S., Erven K., Neyts J., Pannecouque C., Vermeyen T., Herrebout W.A., Pieters L., Tuenter E. (2023). A New Alkaloid from *Pancratium Maritimum*—Structure Elucidation Using Computer-Assisted Structure Elucidation (CASE) and Evaluation of Cytotoxicity and Anti-SARS-CoV-2 Activity. Phytochem. Lett..

[51] Taher R.F., Abd El ghany E.M., El-Gendy Z.A., Elghonemy M.M., Hassan H.A., Abdel Jaleel G.A., Hassan A., Sarker T.C., Abd-ElGawad A.M., Farag M.A. (2025). In Vivo Anti-Ulceration Effect of *Pancratium Maritimum* Extract against Ethanol-Induced Rats via NLRP3 Inflammasome and HMGB1/TLR4/MYD88/NF-Κβ Signaling Pathways and Its Extract Metabolite Profile. PLoS ONE.

[52] Melliti M., Horchani M., Alsaiari N.A., Hamdi A., Ben Jannet H., Mastouri M., Hamoudi M., Edziri H. (2024). Microencapsulation of *Pancratium Maritimum* Essential Oil: GC–MS Analysis, Antimicrobial, Antibiofilm and Anticoagulant Effects and Molecular Docking Evaluation. Chem. Afr..

[53] Dzhoglova V., Ivanov K., Karcheva-Bahchevanska D., Koleva N., Bozhkova M., Benbassat N., Ivanova S. (2024). *Salicornia Europaea* L. and *Suaeda Maritima* (L.) Dumort: Bioactive Compounds and Future Perspectives. Biotechnol. Biotechnol. Equip..

[54] Kim S., Lee E.Y., Hillman P.F., Ko J., Yang I., Nam S.J. (2021). Chemical Structure and Biological Activities of Secondary Metabolites from *Salicornia Europaea* L.. Molecules.

[55] Rahmani R., El Arbi K., Aydi S.S., Hzami A., Tlahig S., Najar R., Aydi S., Debouba M. (2022). Biochemical Composition and Biological Activities of *Salicornia Europaea* L. from Southern Tunisia. J. Food Meas. Charact..

[56] Meot-Duros L., Le Floch G., Magné C. (2008). Radical Scavenging, Antioxidant and Antimicrobial Activities of Halophytic Species. J. Ethnopharmacol..

[57] Correia I., Antunes M., Tecelão C., Neves M., Pires C.L., Cruz P.F., Rodrigues M., Peralta C.C., Pereira C.D., Reboredo F. (2024). Nutritive Value and Bioactivities of a Halophyte Edible Plant: *Crithmum Maritimum* L. (Sea Fennel). Plants.

[58] Souid A., Della Croce C.M., Frassinetti S., Gabriele M., Pozzo L., Ciardi M., Abdelly C., Ben Hamed K., Magné C., Longo V. (2021). Nutraceutical Potential of Leaf Hydro-Ethanolic Extract of the Edible Halophyte *Crithmum Maritimum* L.. Molecules.

[59] Nabet N., Boudries H., Chougui N., Loupassaki S., Souagui S., Burló F., Hernández F., Carbonell-Barrachina Á.A., Madani K., Larbat R. (2017). Biological Activities and Secondary Compound Composition from *Crithmum Maritimum* Aerial Parts. Int. J. Food Prop..

[60] Rossi P.-G., Berti L., Panighi J., Luciani A., Maury J., Muselli A., Serra D.d.R., Gonny M., Bolla J.-M. (2007). Antibacterial Action of Essential Oils from Corsica. J. Essent. Oil Res..

[61] Landoulsi A., Hennebelle T., Bero J., Rivière C., Sahpaz S., Quetin-Leclercq J., Neut C., Benhamida J., Roumy V. (2020). Antimicrobial and Light-Enhanced Antimicrobial Activities, Cytotoxicity and Chemical Variability of All Tunisian Eryngium Species. Chem. Biodivers..

[62] Tóth B., Liktor-Busa E., Urbán E., Csorba A., Jakab G., Hohmann J., Vasas A. (2016). Antibacterial Screening of Juncaceae Species Native to the Carpathian Basin against Resistant Strains and LC-MS Investigation of Phenanthrenes Responsible for the Effect. Fitoterapia.

[63] Sahli R., Rivière C., Neut C., Bero J., Sahuc M.-E., Smaoui A., Beaufay C., Roumy V., Hennebelle T., Rouillé Y. (2017). An Ecological Approach to Discover New Bioactive Extracts and Products: The Case of Extremophile Plants. J. Pharm. Pharmacol..

[64] Iltaf M., Niaz S.I., Majeed M.K., Saleem M., Shah M., Ali M., Shakeel Abbas S., Amin A. (2025). DFT, GC-MS Analysis and Biological Evaluation of *Limbarda Crithmoides* L. Dumort Essential Oil; an Important Edible Halophyte Grown in Pakistan. Nat. Prod. Res..

[65] Tosun F., Kızılay Ç., Şener B., Vural M., Palittapongarnpim P. (2004). Antimycobacterial Activity of Some Turkish Plants. Pharm. Biol..

[66] Paschoalinotto B.H., Polyzos N., Pires T.C.S.P., Molina A.K., Mandim F., Prieto M.A., Dias M.I., Barros L., Petropoulos S.A. (2026). Biochemical Profile and Biofunctional Effects of *Crithmum Maritimum* L. from Three Harvests under Different N:P:K Ratios. Front. Nutr..

[67] Kikowska M., Kalemba D., Dlugaszewska J., Thiem B. (2020). Chemical Composition of Essential Oils from Rare and Endangered Species—*Eryngium Maritimum* L. and E. *Alpinum* L.. Plants.

[68] Elkiran O., Avsar C., Veyisoglu A., Bagci E. (2023). The Chemical Composition and Biological Activities of Essential Oil from the Aerial Parts of *Eryngium Maritimum* L. (Apiaceae). J. Essent. Oil Bear. Plants.

[69] Kovács A., Vasas A., Hohmann J. (2008). Natural Phenanthrenes and Their Biological Activity. Phytochemistry.

[70] Xiong J., Li S., Wang W., Hong Y., Tang K., Luo Q. (2013). Screening and Identification of the Antibacterial Bioactive Compounds from *Lonicera Japonica* Thunb. Leaves. Food Chem..

[71] Stanković M., Stojanović-Radić Z., Jakovljević D., Zlatić N., Luković M., Dajić-Stevanović Z. (2023). Coastal Halophytes: Potent Source of Bioactive Molecules from Saline Environment. Plants.

[72] Hetta M.H., Shafei A.A. (2013). Comparative Cytotoxic and Antimicrobial Activities of the Alkaloid Content of Egyptian *Pancratium Maritimum L.* Fruits and Flowers. J. Am. Sci..

[73] Limongelli F., Aresta A.M., Tardugno R., Clodoveo M.L., Barbarossa A., Carocci A., Zambonin C., Crupi P., Panić M., Corbo F. (2025). Exploitation of Apulian *Salicornia Europaea* L. via NADES-UAE: Extraction, Antioxidant Activity and Antimicrobial Potential. Molecules.

[74] Gonzalez-Pastor R., Carrera-Pacheco S.E., Zúñiga-Miranda J., Rodríguez-Pólit C., Mayorga-Ramos A., Guamán L.P., Barba-Ostria C. (2023). Current Landscape of Methods to Evaluate Antimicrobial Activity of Natural Extracts. Molecules.

[75] Kuete V. (2010). Potential of Cameroonian Plants and Derived Products against Microbial Infections: A Review. Planta Med..

[76] Marongiu B., Maxia A., Piras A., Porcedda S., Tuveri E., Gonçalves M.J., Cavaleiro C., Salgueiro L. (2007). Isolation of *Crithmum Maritimum* L. Volatile Oil by Supercritical Carbon Dioxide Extraction and Biological Assays. Nat. Prod. Res..

[77] Alves-Silva J.M., Guerra I., Gonçalves M.J., Cavaleiro C., Cruz M.T., Figueirinha A., Salgueiro L. (2020). Chemical Composition of *Crithmum Maritimum* L. Essential Oil and Hydrodistillation Residual Water by GC-MS and HPLC-DAD-MS/MS, and Their Biological Activities. Ind. Crops Prod..

[78] Thiem B., Goślińska O., Kikowska M., Budzianowski J. (2010). Antimicrobial Activity of Three Eryngium L. Species (Apiaceae). Herba Pol..

[79] Bucchini A., Ricci D., Messina F., Marcotullio M.C., Curini M., Giamperi L. (2015). Antioxidant and Antifungal Activity of Different Extracts Obtained from Aerial Parts of *Inula Crithmoides* L.. Nat. Prod. Res..

[80] Omezzine F., Daami-Remadi M., Rinez A., Ladhari A., Haouala R. (2019). In Vitro Assessment of *Inula* spp. Organic Extracts for Their Antifungal Activity against Some Pathogenic and Antagonistic Fungi. Afr. J. Microbiol. Res..

[81] Sur-Altiner D., Gurkan E., Mutlu G., Tuzlaci E., Ang O. (1999). The Antifungal Activity of *Pancratium Maritimum*. Fitoterapia.

[82] Cabral C., Cavaleiro C., Gonçalves M.J., Cruz M.T., Lopes M.C., Salgueiro L. (2013). *Otanthus Maritimus* (L.) Hoffmanns. & Link as a Source of a Bioactive and Fragrant Oil. Ind. Crops Prod..

[83] Fu Y., Sarkar P., Bhunia A.K., Yao Y. (2016). Delivery Systems of Antimicrobial Compounds to Food. Trends Food Sci. Technol..

[84] Oulahal N., Degraeve P. (2022). Phenolic-Rich Plant Extracts With Antimicrobial Activity: An Alternative to Food Preservatives and Biocides?. Front. Microbiol..

[85] Zhou X., Zeng M., Huang F., Qin G., Song Z., Liu F. (2023). The Potential Role of Plant Secondary Metabolites on Antifungal and Immunomodulatory Effect. Appl. Microbiol. Biotechnol..

[86] Lagrouh F., Dakka N., Bakri Y. (2017). The Antifungal Activity of Moroccan Plants and the Mechanism of Action of Secondary Metabolites from Plants. J. Mycol. Med..

[87] Ullah F., Guru-Pirasanna-Pandi G., Murtaza G., Sarangi S., Gul H., Li X., Chavarín-Gómez L.E., Ramírez-Romero R., Guedes R.N.C., Desneux N. (2025). Evolving Strategies in Agroecosystem Pest Control: Transitioning from Chemical to Green Management. J. Pest Sci..

[88] Plamada D., Vodnar D.C. (2022). Polyphenols—Gut Microbiota Interrelationship: A Transition to a New Generation of Prebiotics. Nutrients.

[89] Maoloni A., Cardinali F., Milanović V., Osimani A., Verdenelli M.C., Coman M.M., Aquilanti L. (2022). Exploratory Study for Probiotic Enrichment of a Sea Fennel (*Crithmum Maritimum* L.) Preserve in Brine. Foods.

[90] Abd El-Hack M.E., Samak D.H., Noreldin A.E., Arif M., Yaqoob H.S., Swelum A.A. (2018). Towards Saving Freshwater: Halophytes as Unconventional Feedstuffs in Livestock Feed: A Review. Environ. Sci. Pollut. Res..

[91] Oliveira M., Pereira C.G., Castañeda-Loaiza V., Rodrigues M.J., Neng N.R., Hoste H., Ben Hamed K., Custódio L. (2025). Seasonal Biochemical Variations in Mediterranean Halophytes and Salt-Tolerant Plants: Targeting Sustainable Innovations in Ruminant Health. Appl. Sci..

